# Patient Organizations’ Funding from Pharmaceutical Companies: Is Disclosure Clear, Complete and Accessible to the Public? An Italian Survey

**DOI:** 10.1371/journal.pone.0034974

**Published:** 2012-05-09

**Authors:** Cinzia Colombo, Paola Mosconi, Walter Villani, Silvio Garattini

**Affiliations:** 1 Laboratory of Medical Research on Consumer Involvement, Department of Oncology, Istituto di Ricerche Farmacologiche Mario Negri, Milano, Italy; 2 Istituto di Ricerche Farmacologiche Mario Negri, Milano, Italy; University of British Columbia, Canada

## Abstract

**Background:**

Many patients’ and consumers’ organizations accept drug industry funding to support their activities. As drug companies and patient groups move closer, disclosure become essential for transparency, and the internet could be a useful means of making sponsorship information accessible to the public. This survey aims to assess the transparency of a large group of Italian patient and consumer groups and a group of pharmaceutical companies, focusing on their websites.

**Methodology/Principal Findings:**

Patient and consumer groups were selected from those stated to be sponsored by a group of pharmaceutical companies on their websites. The websites were examined using two forms with principal (name of drug companies providing funds, amount of funding) and secondary indicators of transparency (section where sponsors are disclosed, update of sponsorship). Principal indicators were applied independently by two reviewers to the patient and consumer groups’ websites. Discordances were solved by discussion. One hundred fifty-seven Italian patient and consumer groups and 17 drug companies were considered. Thirteen drug companies (76%) named at least one group funded, on their Italian websites. Of these, four (31%) indicated the activities sponsored and two (15%) the amount of funding. Of the 157 patient and consumer groups, 46 (29%) named at least one pharmaceutical company as providing funds. Three (6%) reported the amount of funding, 25 (54%) the activities funded, none the proportion of income derived from drug companies. Among the groups naming pharmaceutical company sponsors, 15 (33%) declared them in a dedicated section, five (11%) on the home page, the others in the financial report or other sections.

**Conclusions/Significance:**

Disclosure of funds is scarce on Italian patient and consumer groups’ websites. The levels of transparency need to be improved. Disclosure of patient and consumer groups provided with funds is frequent on Italian pharmaceutical companies’ websites, but information are often not complete.

## Introduction

Patient and consumer groups are increasingly considered an important point of reference by healthcare agencies, research institutions, medical societies and the drug industry, thanks to their role and work for patients. Different aims and interests are at stake in working with patient groups, such as involving patients in research projects or clinical studies, consulting patients in advisory committees or boards, conveying information to patients, lobbying regulatory agencies and institutions.

As regards drug companies, some of their interests are to boost knowledge about patients’ needs, improve their image supporting patient groups as a corporate responsibility activity, and putting pressure on prescribers and regulatory agencies.

Many patient and consumer groups accept drug industry funding to support their activities. Some of them see this as the only way to reach their aims and efficiently respond to patients’ demands [1] - especially considering the lack of public funds - relying on the capacity of patients groups to defend their independence from the influence of any sponsor. [1], [2] Accepting funding from the drug industry clearly puts patient organizations in a condition of potential conflict of interest where their independence and public trust are at risk, considering the influence of drug industry funding on sponsored studies or activities. [3]–[4] Patient groups can unintentionally promote a drug or a diagnostic test towards patients- acting as a neutral third party –they can overlap drug companies’ and patients’ interests in the policy of their organization; finally, they can be prevented from making autonomous decisions, especially when patients’ interests differ from those of the drug industry. [1]–[2], [5]–[6] Awareness of these risks and the development of policies to manage them are not common among patient groups and, even if the situation varies in different countries, the need to disclose funding to the public has not been adequately addressed by patient groups. [7], [8] As drug companies and patient groups move closer, disclosure and openness become essential for transparency, and the internet could be a useful means of making sponsorship information accessible to the public.

Transparency of patient groups in different countries has been assessed, [7], [9]–[12] but Italian data are rare. [8] The Laboratory of Medical Research on Consumer Involvement at Mario Negri Institute own conducted surveys in Italy on convenience samples of patient groups’ websites (in 2008, 2009). The results suggested a low level of transparency regarding funding from the drug industry: only a few websites declared the funding and published a code of conduct about sponsorship. Data were presented and discussed with a network of patient groups during training courses promoted by the Laboratory (data not published). Data and information must be publicly available for transparency and the internet offers a good way of meeting this requirement. The present study assessed the transparency of a large group of Italian patient and consumer groups and a group of pharmaceutical sponsor companies, through defined indicators, focusing on the information reported on their websites.

## Methods

Firstly, the pharmaceutical companies were selected on the basis of their market sales, then their websites were searched for listings of patient groups – dealing with specific disease – and consumer groups – dealing with healthcare rights, quality of healthcare services, topics of public interest (such as screening programs, awareness campaigns).

Patient and consumer groups were then selected to obtain the final sample and, finally, the pharmaceutical companies’ and the patient and consumer groups’ websites were assessed.

### Selection of Pharmaceutical Companies

The drug companies were selected among the top fifteen global corporations for sales in 2009, [13] adding a group of Italian companies we considered important for the Italian market (Text S1).

The Italian and the international websites of each drug company – if available – were visited on 10 and 31 March 2010, to assess their transparency and select Italian patient and consumer groups funded. This information was collected by searching in sections called “patient associations”, “collaborations”, “corporate responsibility”, etc.

### Selection of Patient and Consumer Groups

Patient and consumer groups were selected considering the most recent year of sponsorship available on pharmaceutical companies’ websites. Groups dealing only with social services and those strictly related to healthcare professionals or hospitals (i.e. funded by clinicians, or exclusively dedicated to fund raising for hospital departments) were excluded.

Two researchers (CC, PM) independently searched for the websites of selected patient and consumer groups on Google (on 13 and 29 April 2010), using as search terms first the name of the group, then the name of the disease (for example diabetes, cancer) or area of interest (for example consumers healthcare). If the website was not found, the group was excluded from the survey. Additional information on these groups were sought by mail.

### Evaluation

The transparency of websites of patient and consumer groups and pharmaceutical companies was assessed, in terms of: disclosure of sponsorship, amount of funds, activities funded, accessibility of sponsorship information, code of conduct about sponsorship, links to other websites. Disclosure information was compared between the company providing fund and each group funded.

#### Patient and consumer groups

Patient and consumer groups’ websites were examined using a defined form based on a previous published form. [7] A preliminary version was developed taking account of the suggestions made by a group of patients’ representatives during a training course dealing with conflicts of interest [14]: questions 16 and 21 were added (Text S2).

The final version of the form was pilot-tested on a random sample of 47 websites (about 30% of the total websites) independently evaluated by two reviewers (CC, PM). Discordances were solved by discussion (July 2010). Concordance on the main indicator considered, “Drug industries providing fund to the group are disclosed in the website”, was good (82%). Discussion of discordances led to strict definitions of the criteria to be applied during the data collection. After the resolution of discordances, the data of the websites evaluated in the pilot test were included in the final analysis. The form is divided into general indicators of transparency, main and secondary indicators related to funding received from drug companies (Text S2).

Two reviewers (CC, PM) independently assessed the general indicators and the main indicators of transparency related to funding received from pharmaceutical companies (websites visited on May, June, Sept 2010). Discordances were solved by discussion. The secondary indicators were evaluated by one reviewer (October 2010).

Transparency and disclosure information were also evaluated according to the disease of interest and the area of activity of the patient and consumer groups included. The area of activity was defined according to the statute of each group – where available – and the information about activities and projects reported on the website.

Disclosure practices of patient and consumer groups excluded from the survey for the lack of a website were also explored. Their mail addresses or e-mails were searched in the Laboratory’s database of patient and consumer groups, the list of volunteers’ groups issued by the Italian Revenue Agency, the search engine Google. They were contacted once, by mail or e-mail, with a short description of the study and three questions about their disclosure practices (presence of a code of conduct about sponsorships, description of the norms…).

#### Pharmaceutical companies

The transparency of pharmaceutical companies’ websites about funding provided to patient and consumer groups was assessed using a form defined on the basis of previous studies [7], [15]. It is divided into main and secondary indicators of transparency (Text S3). One reviewer applied the form to the Italian websites of the 17 drug companies included. Some indicators were compared on the Italian and international website of each pharmaceutical company. Disclosure information available on the websites of pharmaceutical companies based in Italy was compared to that available on the websites of pharmaceutical companies based abroad.

## Results

**Figure 1:**
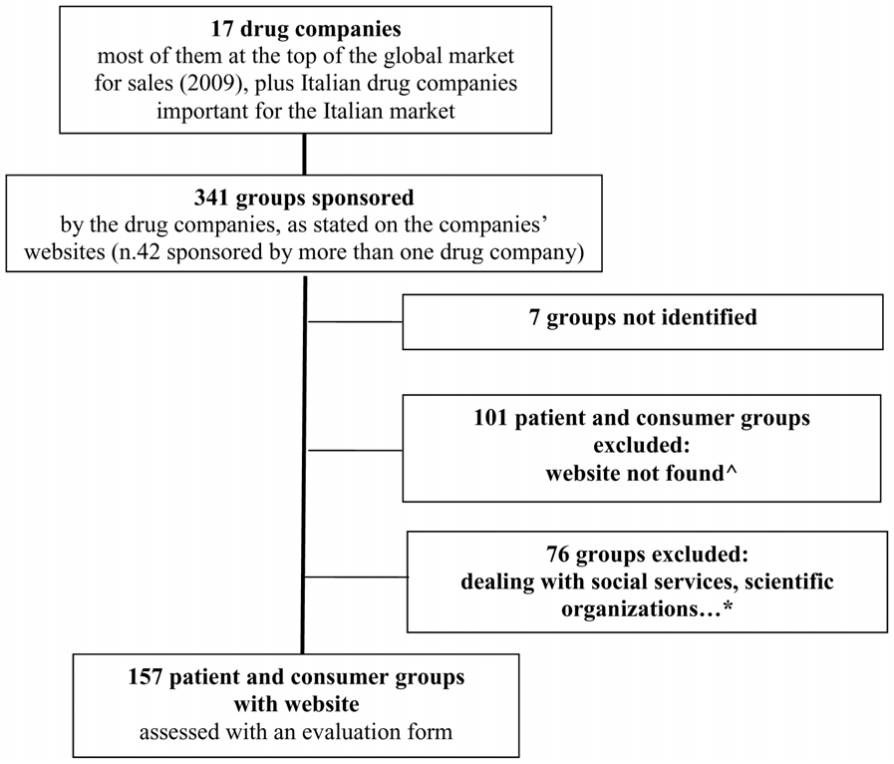
Flow chart. The drug companies were selected among the top fifteen global corporations for sales in 2009, adding a group of Italian companies important on the Italian market. Their websites were searched for listings of patient and consumer groups funded. Groups dealing only with social services and strictly related to healthcare professionals or hospitals were excluded. The websites of selected patient and consumer groups were searched on Google. If the website was not found, the group was excluded from the survey. **^∧^** Additional information of groups with no website were sought by mail. * 76 groups not responding to the inclusion criteria: 33 dealing with social services or supporting sports or social activities. 38 scientific organizations, or dedicated to fundraising for medical research, or hospital department, or mainly composed of healthcare operators. 5 groups based in countries other than Italy (listed among the Italian organizations).

In all, 17 pharmaceutical companies were selected. Five have their headquarter in Italy, 12 abroad (Table 1). At the time of the survey 13 (76%) named at least one patient or consumer group funded, for a total of 341 groups. The sponsorships referred to the years 2008, 2009, 2010, considering the date of update available (8 websites). Groups listed in these sections included scientific societies, associations supporting medical research, and groups dealing with social services. Seven were not identified. Applying the selection criteria to the 334 groups identified, 177 (53%) groups in all were excluded from the survey, 101 (30%) for the lack of a website (Figure 1). One hundred and fifty-seven groups (47%) were included in the survey (Table 2). These groups are mainly based in the North of Italy (48%), with a mean age of 19.6 years (range 2–89). Groups on diabetes are the most common, followed by groups on cancer, hematology, neurodegenerative diseases, autoimmune diseases, cardiovascular diseases, AIDS HIV, transplants, behavioral disorders, respiratory diseases, other diseases (such as growth hormone deficiency, psoriasis…) and consumer groups.

**Table 1:** Main details of the 17 pharmaceutical companies.

Headquarter based in	n. (%)
Italy	5 (29)
Other European countries	7 (41)
United States	5 (29)
**Annual revenue 2009,** EU billion	**mean; range**
All	19.5; 0.5–43.1
Italian headquarter (n.5)	1.2; 0.5–2.8

Description of drug companies included in the survey: headquarter, annual revenue (2009) for all the drug companies and the Italian ones (mean and range).

**Table 2:** Main details of 157 patients and consumers groups.

Base	n. (%)
Northern Italy	76 (48)
Central Italy	55 (35)
Southern Italy	26 (17)
**Area of activity**	
National	49 (31)
Regional	29 (18)
Provincial	63 (40)
Local	16 (10)
**Years from foundation**	**mean; range**
Data available for 139 (88%) organizations’ websites	19.6; 2–89
**N. of members**	**mean; range**
Data available for 51 (32%) organizations’ websites	
Organizations/groups*	23; 17–85
Sections**	47; 4–300
Individual members**	2,204; 17–13,930
**Disease or area of interest**	
diabetes	49 (31)
cancer	24 (15)
hematology	21 (13)
neurodegenerative diseases	12 (8)
autoimmune diseases	10 (6)
AIDS/HIV	6 (4)
cardiovascular diseases	5 (3)
transplants	5 (3)
behavioral disorders	4 (2)
respiratory diseases	2 (1)
other diseases ∧	12 (8)
consumer groups	7 (4)

* federations or coalitions (comprising many organizations).

** for single organizations the number of sections (local units) is reported if available on the website, otherwise the number of volunteers/individuals is given.

∧ organizations not included in other categories: e.g. growth hormone deficiency, psoriasis, inflammatory bowel disease, osteoporosis, prevention of blindness.

Description of patient and consumer groups included in the survey: headquarter, main area of activities (going from the larger areas – i.e. regions – to the smaller ones – i.e. single cities or municipality), age (mean and range**),** number of members (mean and range) classified as: number of groups (for coalitions or federations), number of sections or local units (for single organizations), number of individuals (for single organizations); disease of interest.

### Transparency of Patient and Consumer Groups’ Websites

More than a quarter of the websites (n. 46, 29%) named at least one pharmaceutical company as providing funding (Table 3). Of these, three (6%) reported the amount of funding received, 25 (54%) named activities funded, none reported the proportion of income derived from the drug industry. Fifteen groups disclosed the name of drug companies in a dedicated section (33% of those disclosing), 5 (11%) on the home page, the others only in the financial report (n. 5, 11%) or in other sections not specifically dedicated to sponsors (n. 21, 45%). As a result, the availability of information about sponsorships varied widely on the websites. The date of update of sponsorships was reported by 29 groups (63% of those disclosing the drug companies sponsors), nine (less than 20%) stated that the funding was unrestricted.

**Table 3:** Transparency of 157 patient and consumer groups’ websites.

	All	Diabetes	Cancer	Hematology	Neurodegenerativediseases	Autoimmunediseases	Cardiovasculardiseases	AIDSHIV	Transplants	Behavioraldisorders	Respiratorydiseases	Others*	Consumergroups
	157	49	24	21	12	10	5	6	5	4	2	12	7
	n (%)	n (%)	n. (%)	n (%)	n (%)	n (%)	n (%)	n (%)	n (%)	n (%)	n (%)	n (%)	n (%)
**Disclosure of drug companies providing funds**	46 (29)	15 (31)	7 (29)	3 (14)	3 (25)	2 (20)	2 (40)	2 (33)	0	1 (25)	2 (100)	4 (33)	5 (71)
disclosure in a dedicated sectionor home page	20 (43)	4 (27)	6 (86)	1 (33)	1 (33)	1 (50)	1 (50)	0	-	0	0	1 (25)	5 (100)
disclosure of amount of funding	3 (6)	1 (7)	0	1 (33)	0	0	0	1 (50)	-	0	0	0	0
disclosure of activities funded**	25 (54)	9 (60)	1 (14)	2 (67)	2 (67)	1 (50)	1 (50)	2 (100)	-	1 (100)	1 (50)	3 (75)	2 (29)
* core activities*°	10 (40)	2 (22)	1 (100)	1 (50)	1 (50)	0	1 (100)	1 (50)		0	0	1 (33)	2 (100)
* educational activities*°	22 (88)	7 (78)	1 (100)	1 (50)	2 (100)	1 (100)	1 (100)	2 (100)		1 (100)	1 (100)	3 (100)	2 (100)
* research activities*°	7 (28)	3 (33)	0	0	1 (50)	0	0	1 (50)		0	0	0	2 (100)
**Code of conduct dealing with sponsorships**	14 (9)	2 (4)	2 (8)	0	2 (22)	0	1 (20)	1 (33)	0	0	2 (100)	0	4 (57)
**Link to drug companies’** **websites or sponsored by drug** **companies**	42 (27)	27 (55)	3 (12)	1 (5)	2 (22)	1 (10)	0	2 (67)	0	0	2 (100)	2(17)	2 (29)
**Banner advertising in home** **page:**	68 (43)	27 (55)	6 (25)	8 (38)	9 (75)	3 (30)	1 (20)	3 (50)	3 (60)	0	0	6 (50)	2 (29)
Editorial content clearlyseparated from advertising	54 (79)	22 (81)	4(67)	7 (88)	7 (78)	1(33)	1 (100)	2 (67)	3 (100)	-	-	5(83)	2 (100)
**Financial report available**	30 (19)	5 (10)	6 (25)	7 (33)	3 (25)	1 (10)	2 (40)	2 (33)	0	0	1 (50)	1 (8)	2 (29)

* organizations not included in other categories: e.g. growth hormone deficiency, psoriasis, inflammatory bowel disease, osteoporosis, prevention of blindness.

** reference varies among organizations: some describe the activities funded in detail, others only mention them. Some report this information only in brochures of events or activities.

°percentages refer to the organizations reporting funded activities. More than one answer was possible.

The transparency of patient and consumer groups’ websites was assessed in terms of: disclosure of drug companies providing funds, amount of funds, activities funded; accessibility of sponsorship information; availability of a code of conduct about sponsorship; links to drug companies’ websites; presence of banner advertisings; clear separation of marketing content; availability of a financial report.

Disclosure information are reported for all the groups and by disease of interest.

Drug industry logos were used in 26 websites (17% of all groups), banner advertisements of products (drugs, medical devices or booklets made by drug companies) were reported by 17 websites (about 11%), 42 (27%) had links to drug industry websites or strictly related to drug industries.

A financial report was published by 30 groups (19% of all groups). Of these, sixteen (53%) were updated to 2009, the others were not updated. The number of members (individuals or corporate members, respectively of single groups or federations) were not reported by 106 groups (67%); 68 websites (43%) displayed banner advertising (any commercial advertising, not only of drug companies’ products) on the home page; most of them (80%) clearly separated the editorial content from advertising. Editorial policy – a clear statement describing what procedure is used for selecting content- was hardly ever explained (only 2%). Table 3 summarizes the findings.

#### Transparency by disease and area of activity

Patient and consumer groups were classified by disease and area of activity to assess the transparency of websites. Groups on diabetes (n.49, 31%) are the most common type of patient group represented in the sample, followed by groups on cancer (n. 24, 15%), hematology (n.21, 13%) and neurodegenerative diseases (n.12, 8%).

Table 3 lists some indicators of transparency by disease of interest. Differences should be considered in relation to the number of groups per disease, and the selection criteria applied. Taking these limits into account, for some disease there is a large number of websites publishing banner advertisements, links to drug company websites, or sites related to drug companies, and, at the same time, only a few websites publishing financial reports, disclosing drug industry sponsorships, or publishing codes of conduct about sponsors. For example, 31% of groups on diabetes disclosed pharmaceutical companies providing funds, 55% published banner advertising, 55% linked to drug companies’ websites or websites sponsored by drug companies, 10% published the financial report, 4% a code of conduct dealing with sponsorship. Twenty-nine per cent of groups on cancer disclosed pharmaceutical companies providing funds, 12% linked to drug companies’ websites or websites sponsored by drug companies, 25% published the financial report and 8% a code of conduct. Fourteen per cent of hematology groups disclosed pharmaceutical companies providing funds, 5% linked to drug companies’ websites or websites sponsored by drug companies, 33% published the financial report and no one published a code of conduct.

Groups were also divided into two groups according to their area of activity: nationwide and regional or province and local areas. National and regional groups were more likely to disclose sponsorships from the pharmaceutical industry and to publish codes of conduct than the provincial and local ones (significant results; data not shown).

### Transparency of Drug Companies’ Websites

According to the patient groups’ websites disclosing the drug companies providing sponsorships, each of the selected drug companies supported at least one of the patient or consumer groups included in the survey (one through its foundation).

Thirteen of the 17 drug companies (76%) named at least one patient or consumer group funded, on their Italian websites. Four indicated the projects or activities sponsored and two the amount of funding (Table 4). Reference to the activities sponsored varied for drug companies, from detailed description to generic mention of the type of activity (e.g. education, information, advocacy).

**Table 4:** Disclosure information on 17 pharmaceutical companies’ websites providing funds to patient and consumer organizations.

Principal indicators	n (%)
Disclosure of the name of at least one patient or consumer organization funded∧	13 (76)
If yes, the amount of funding is reported	2 (15)
If yes, activities funded are reported*	4 (31)
**Secondary indicators**	
Funded patient and consumer organizations are disclosed:**	
in a dedicated section	11 (85)
in other sections	2 (15)
Date of update of sponsorships is available**	8 (61)
Links to websites of funded patient and consumer organizations**	5 (38)
Code of conduct dealing with patients and consumers organizations	15 (88)
An international website	13 (76)
If yes, information about sponsorship is different from that on the Italian website	9 (77)

∧ March 2010.

* Reference to the activities funded varied among drug companies, from detailed description to generic mention of the type of activity.

** Percentages refer to the drug companies reporting patient and consumer organizations funded.

The transparency of drug companies’ websites was assessed in terms of: disclosure of patient and consumer groups funded, amount of funds, activities funded; accessibility of sponsorship information; date of update of sponsorship; links to websites of funded patient and consumer groups; availability of a code of conduct on the relationships with patient and consumer groups; different sponsorships information between the international and the Italian website of the drug company.

According to the patient groups’ websites, each of the selected drug companies supported at least one of the patient or consumer groups included in the survey (one through its foundation).

Among the 13 drug industry websites disclosing sponsorships, 11 (85%) declared them in a dedicated section (as “patient associations”, “collaborations”, “grants”, “corporate responsibility”, etc.), the others reported them in different areas covering for example specific projects. Five websites (38% of those declaring sponsorships) had links to sponsored patient and consumer groups websites. Fifteen drug companies (88%) published codes of conduct dealing with sponsorship, on their websites (Table 4).

#### Disclosure information available on the Italian pharmaceutical companies websites and others

Thirteen drug companies had an international website as well as the Italian one. Nine gave different information on the Italian website and the international one for at least one of the indicators of transparency considered. Four declared on the international website that they supported Italian patient or consumer group different in number or name from those declared in the Italian website. Five reported the sponsored projects only in the international website, three the amount of funding.

Comparing the five pharmaceutical companies based in Italy and the 12 based abroad, respectively 3 (60%) and 10 (83) named at least one patient and consumer group provided with funding. None of the Italian pharmaceutical companies reported the projects funded, or the amount of funding.

### Information about Funding Reported on Pharmaceutical Companies’ Websites and Patient and Consumer groups’ Websites

Correspondence was low between sponsorships disclosed by drug companies and by patient consumer groups. All the patient and consumer groups received funding from the drug industry - according to the information available on pharmaceutical companies websites - however, only 46 (29%) stated they received funding, for a total of 162 sponsorships from drug companies (the range of drug companies sponsors for single group was 1–11). Thirty patient and consumer groups (65%) stated at least one drug company corresponding to the disclosure made by the drug company itself. The other groups (35%) declare other drug companies as sponsors. A fourth of the 162 sponsorships disclosed corresponded to the disclosure made by the 13 drug companies considered (data not shown). On the other side, each drug company supported at least one patient or consumer group, but only 13 mentioned it.

### Patient and Consumer Groups with no Website

Eighty-five groups (84% of 101)– whose addresses (mail or e-mail) were found – were contacted (January 2011). Three (4%) responded to our questions about their disclosure practices: two had no code of conduct about sponsors, one had a code but did not specify the norms or give us examples of its application.

## Discussion

### Transparency of Patient and Consumer Groups

The patient and consumer groups with websites selected for this survey cover a wide range for disease of interest, number of members and area of activity. The collected data refer to Italy, even so the findings are relevant also to other countries.

The disclosure of sponsorships on patient and consumer groups’ websites is scant and poorly accessible. Only 13% of all the groups disclosed funding from pharmaceutical companies in a easily accessible area, the others solely in the financial report or in sections dealing with single activities, often on brochures or posters of sponsored events.

Few patient and consumer groups declared the type of activity funded (16% on all the groups). Educational activities, including spread of information and meetings, were the most funded. The date of update of sponsorships was lacking on a third of the groups’ websites declaring sponsorships and the level of transparency was also low for financial reports.

It was impossible to quantify the funding received by each patient and consumer group, as the information was only occasionally reported (2% of all the groups included). To give an idea, the two drug companies reporting this data on their websites funded a minimum of 1,500 and a maximum of 90,000 Euros per group (in 2009). It was also impossible to quantify the proportion of funding received by the groups on the total amount of funds, which is an important indicator of their independence from the sponsor. The only drug company reporting this on its website said it funded from less than 5% to almost 30% of the total budget of a single group (in 2009).

The scant attention to disclosure of conflicts of interest is confirmed by the lack of codes of conduct related to sponsors for most of the patient and consumer groups. Previous surveys on disclosure of funding on patient groups’ websites found that most of them did not declare sponsorships from drug industry, with some differences between countries. [7], [12]–[15] According to a survey of 69 websites of patient and consumer groups based in the United States, United Kingdom, Australia, South Africa and other international groups (none in Italy), 45% declared they received funding from the drug industry, none reported the proportion of funding, one third showed drug companies logos and/or links to their websites. [7] A survey on patient groups in the United States and funded by Eli Lilly gave even more alarming results: only a fourth of groups acknowledged the sponsor on their websites. [12].

As the patient and consumer groups based abroad, also the Italian ones do not usually declare the amounts received. According to the results of this survey, patient and consumer groups with a broad area of activity (at national or regional level) are more likely to declare to receive funding from the drug industry.

### Transparency of Drug Company Websites

Most of the websites of Italian drug manufactures declared they sponsored at least one patient and consumer group. Availability of information was very variable and only few companies fully disclosed sponsorships: only four (Pfizer, Novartis, GSK, MSD) indicated the patient and consumer groups’ activities funded, two the amount of funding (Pfizer, GSK). Indirect support was not reported - for example financial support for participation in training courses. Sponsorship information available on Italian websites was generally less complete than on the international websites.

Considering both Italian and international websites, about half the drug companies reported funded projects. Less than a third reported the amount of funding. All except two published codes of conduct dealing with patient and consumer groups.

The policy on disclosure of sponsorships varies according to the legislation of the country where a company is based and the national or international codes of conduct followed by the drug industry. Different codes of conduct deal with this issue. The code of the European Federation of Pharmaceutical Industries and Associations (EFPIA) [16] adopted by the EFPIA board on 5 October 2007 states that each drug company must make public a list of patient groups to which it provides financial support and/or significant indirect/non-financial support. This should include a short description of the nature of the support. This information may be provided on a national or European level and should be updated at least once a year. The Association of the British Pharmaceutical Industry [17] complies with the EFPIA code, specifying that companies must make public a list of all groups to which they provide support by means of information on their websites or in their annual reports. Farmindustria - an association of all the Italian drug companies included in the survey – also complies with the EFPIA code and specify that companies must make public on their websites a list of all groups to which they provide support. [18] The date of the code was September 2009. Finally, the Association of Voluntary Self-Regulation for the Pharmaceutical Industry (FSA, German) demands public disclosure of the amount of funding given to patient and consumer groups, for the year and the group supported (date of the code: June 2008). [19] Some requirements of the reference codes of conduct were not met by some of the drug companies in this study, at the time of the survey. Among the Italian websites of the 17 drug companies selected, 4 did not disclose any patient groups funded, even if each of them supported at least one of the patient or consumer groups included in the survey. Among the 13 international websites of the drug companies selected, only five reported the sponsored projects, three the amount of funding.

Strict control on the implementation of the codes of conduct by EFPIA, Farmindustria, FSA could be useful to improve the drug companies’ disclosure practices. Finally, governmental agencies and other interested stakeholders should require a public, understandable and detailed disclosure of funding to patient and consumer groups from drug companies and monitor the transparency of information reported on the drug companies websites.

This survey has some limits. First, some of the discrepancies between drug companies’ and patients’ websites could be explained because they may not relate to the same period of time, also because websites, in particular sections reporting sponsorships, were not all updated regularly.

Second, among the patient and consumer groups funded by the drug companies included in the sample, many had not a website. To confirm the lack of a website and to explore their disclosure practices, these groups were contacted once by mail or e-mail with few questions on sponsorships. Only three groups responded, so it was not possible to collect data on the disclosure practices of these groups.

### Conclusions

Transparency in the relationships between patient and consumer groups and drug companies is essential for the credibility of both. It is also necessary for patient, consumers and other stakeholders to critically appraise the messages, demand or proposals from patient and consumer groups and assess which best represents the patients’ point of view.

Complete and accessible information about sponsorships on drug industries’ websites is needed (including the amount of funding for each patient and consumer group, direct and indirect support, the activities supported) and the codes of conduct should be stricter than the current ones, in line with the disclosure requirements for support to health professionals. [20].

This regards holds for patient and consumer groups. As they lie at the center of many interests, they should boost their autonomy and their independence from sponsors. Some refuse drug industry funding in order to maintain their autonomy, others accept it under certain conditions. Considering that undue pressure and influence can come even from other sponsors, such as public agencies, medical societies or research institutes, relationships with sponsors and common policies to maintain independence should be discussed by the patient groups themselves. Even if some Italian patient groups have policies and codes of conduct for these issues, the levels of transparency, disclosure and ability to manage relationships with sponsors need to be improved.

Many patient and consumer groups do not have a website. Considering the increasing use of internet by patients and consumers searching for healthcare information, [21] they should consider to create their own website, in order to strength their role and increase their transparency towards the public.

Patient and consumer groups’ websites should be clearer and more accessible, they should dedicate a section for sponsorships, declaring the amount of funding received and the activities funded.

## Supporting Information

Text S1 **Drug companies included in the survey.**
(DOC)

Text S2 **Form assessing the transparency of patient and consumer groups’ websites.**
(DOC)

Text S3 **Form assessing the transparency of drug companies’ websites about funding provided to patient and consumer groups.**
(DOC)

## References

[1] Kent A (2007). Should patient groups accept money from drug companies? Yes.. BMJ.

[2] Herxheimer A (2003). Relationships between the pharmaceutical industry and patients’ organizations.. BMJ.

[3] Kjaergard LL, Als-Nielsen B (2002). Association between competing interests and authors’ conclusions: epidemiological study of randomised clinical trials published in the *BMJ.*. BMJ.

[4] Lexchin J, Bero LA, Djulbegovic B, Clark O (2003). Pharmaceutical industry sponsorship and research outcome and quality: systematic review.. BMJ.

[5] Yamey G (2000). Drug companies seek MS patients to lobby for new products.. BMJ.

[6] Chalmers I (2007). The Alzheimer’s Society, drug manufacturers, and public trust.. BMJ.

[7] Ball DE, Tisocki K, Herxheimer A (2006). Advertising and disclosure of funding on patient organisation websites: a cross-sectional survey.. BMC Public Health.

[8] Mosconi P (2003). Industry funding of patients’ support groups: Declaration of competing interests is rare in Italian breast cancer associations.. BMJ.

[9] Rothman SM, Raveis VH, Friedman A, Rothman DJ (2011). Health advocacy organizations and the pharmaceutical industry: an analysis of disclosure practices.. American Journal of Public Health.

[10] Hemminki E, Toiviainen HK, Vuorenkoski L (2010). Co-operation between patient organizations and the drug industry in Finland.. Soc Sci Med.

[11] Jones K (2008). In whose interest?.

[12] O’Donovan O (2007). Corporate colonization of health activism? Irish health advocacy organizations’ modes of engagement with pharmaceutical corporations.. Int J Health Serv.

[13] IMS Health Midas (2009). http://www.imshealth.com/deployedfiles/imshealth/Global/Content/StaticFile/Top_Line_Data/Top%20Global%20Corporations_2009.pdf.

[14] Mosconi P, Colombo C (2010). Fostering a strategic alliance between patients’ associations and health care professionals.. J Ambul Care Manage.

[15] Consumers International (2006). http://www.consumersinternational.org/Shared_ASP_Files/UploadedFiles/ECD91B6F-FE37-45C0-.

[16] EFPIA code of conduct (2007). http://www.efpia.eu/Content/Default.asp?PageID=559&DocID=3484.

[17] ABPI code of conduct (2008). http://www.pmcpa.org.uk/?q=patientorganisationsandcode.

[18] Farmindustria code of conduct (2009). http://www.farmindustria.it/pubblico/01cofait.pdf.

[19] IFPMA code of conduct (2008). http://www.ifpma.org/fileadmin/templates/EthicalPromotion/pdfs/Assoc_Code/DE-EN-FSA-Code-Patients-2008.pdf.

[20] Robertson J, Moynihan R, Walkom E, Bero L, Henry D (2009). Mandatory Disclosure of Pharmaceutical Industry-Funded Events for Health Professionals.. 2009;.

[21] Siliquini R, Ceruti M, Lovato E, Bert F, Bruno S (2011). Surfing the internet for health information: an Italian survey on use and population choices.. BMC Med Inform Decis Mak.

