# Supplementary material for: Patient Organizations’ Funding from Pharmaceutical Companies: Is Disclosure Clear, Complete and Accessible to the Public? An Italian Survey
Source: PLoS One. 2012 May 9;7(5):e34974. doi: 10.1371/journal.pone.0034974 (PMC3348919; doi:10.1371/journal.pone.0034974)
Supplement: Text S1 — Drug companies included in the survey. (DOC) [file pone.0034974.s001.doc]

**Text S1. Drug companies included in the survey,** selected among the top fifteen global corporations for sales in 2009, plus a group of Italian companies.

1. Abbott
2. Astrazeneca
3. Bayer
4. Boehringer Ingelheim
5. Bracco
6. Chiesi farmaceutici Spa
7. Dompè
8. GSK
9. Johnson & Johnson
10. Lilly
11. Menarini Firenze
12. Merck & Co -Merck and Schering-Plough
13. Novartis
14. Pfizer Italia
15. Roche
16. Sanofi-Aventis
17. SigmaTau
