# Supplementary material for: Patient Organizations’ Funding from Pharmaceutical Companies: Is Disclosure Clear, Complete and Accessible to the Public? An Italian Survey
Source: PLoS One. 2012 May 9;7(5):e34974. doi: 10.1371/journal.pone.0034974 (PMC3348919; doi:10.1371/journal.pone.0034974)
Supplement: Text S2 — Form assessing the transparency of patient and consumer groups’ websites. (DOC) [file pone.0034974.s002.doc]

**Text S2. Form assessing the transparency of patient and consumer groups’ websites.**

1. Age of the organisation, year of foundation
2. Area of activity
3. Groups/sections/volunteer members

General indicators

1. Advertising banner on the home page
2. Editorial content clearly separated from advertising
3. Clear statement describing the procedure used for selecting content
4. Financial report
5. Date of update of the available financial report

Disclosure of funding received from drug companies

*Principal indicators*

1. Name of drug industries providing funds
2. The amount of funding
3. Activities funded
4. The proportion of funding received on the total budget of the organization

*Secondary indicators*

13. Drug industry sponsors are reported:

a. in a dedicated section or on the home page

b. exclusively in the financial report

c. in other areas, not specifically dedicated to sponsors

14. Date of update of sponsorship is available

15. If yes, specify

16. Funding is related to:

a. *core* activities

b. educational activities

c. research activities

17. Clear statement that funding is unrestricted

18. Code of conduct for dealing with sponsorships

19. Banner advertising of drug companies’ products

20. Drug companies’ logo

21. Links to drug companies’ websites, or websites related to drug companies
