# Supplementary material for: Patient Organizations’ Funding from Pharmaceutical Companies: Is Disclosure Clear, Complete and Accessible to the Public? An Italian Survey
Source: PLoS One. 2012 May 9;7(5):e34974. doi: 10.1371/journal.pone.0034974 (PMC3348919; doi:10.1371/journal.pone.0034974)
Supplement: Text S3 — Form assessing the transparency of drug companies’ websites about funding provided to patient and consumer groups. (DOC) [file pone.0034974.s003.doc]

**Text S3. Form assessing the transparency of drug companies’ websites about funding provided to patient and consumer groups.**

Principal indicators

1. The name of at least one patient or consumer organization funded
2. The amount of funding
3. Activities funded

Secondary indicators

1. Patient and consumer groups funded are reported:

a. in a dedicated section

b. in other sections

1. Date of update of sponsorship is available
2. If yes, specify
3. Links to patient or consumer groups provided with funds
4. Code of conduct for dealing with sponsorships to patient and consumer groups
5. If yes, specify
6. An international website
7. If yes, is the information about sponsorship different from that reported on the Italian website?
8. If yes, specify discordant information :
   1. disclosure of patient and consumer organization funded

b. patient and consumer organizations declared to be funded are different (numbers or names)

c. disclosure of the amount of funding

d. disclosure of the activities funded

e. date of update of sponsorship available

f. links to patient and consumer groups funded

g. code of conduct on sponsorships to patient and consumer groups
